# Renal outcomes in valve‐in‐valve transcatheter versus redo surgical aortic valve replacement: A systematic review and meta‐analysis

**DOI:** 10.1111/jocs.16890

**Published:** 2022-08-30

**Authors:** Arian Arjomandi Rad, Vinci Naruka, Robert Vardanyan, Mohammad Yousuf Salmasi, Panagiotis T. Tasoudis, Simon Kendall, Roberto Casula, Thanos Athanasiou

**Affiliations:** ^1^ Department of Medicine, Imperial College London Faculty of Medicine London UK; ^2^ Department of Cardiothoracic Surgery, Imperial College NHS Trust Hammersmith Hospital London UK; ^3^ Department of Surgery and Cancer Imperial College London London UK; ^4^ Department of Cardiothoracic Surgery University of Thessaly, Biopolis Larissa Greece; ^5^ Department of Cardiothoracic Surgery James Cook University Hospital Middlesborough UK

**Keywords:** acute kidney injury, redo SAVR, renal failure, ViV‐TAVR

## Abstract

**Introduction:**

Postoperative acute kidney injury (AKI) and the requirement for renal replacement therapy (RRT) remain common and significant complications of both transcatheter valve‐in‐valve aortic valve replacement (ViV‐TAVR) and redo surgical aortic valve replacement (SAVR). Nevertheless, the understanding of renal outcomes in the population undergoing either redo SAVR or ViV‐TAVR remains controversial.

**Methods:**

A systematic database search with meta‐analysis was conducted of comparative original articles of ViV‐TAVR versus redo SAVR in EMBASE, MEDLINE, Cochrane database, and Google Scholar, from inception to September 2021. Primary outcomes were AKI and RRT. Secondary outcomes were stroke, major bleeding, pacemaker implantation rate, operative mortality, and 30‐day mortality.

**Results:**

Our search yielded 5435 relevant studies. Eighteen studies met the inclusion criteria with a total of 11,198 patients. We found ViV‐TAVR to be associated with lower rates of AKI, postoperative RRT, major bleeding, pacemaker implantation, operative mortality, and 30‐day mortality. No significant difference was observed in terms of stroke rate. The mean incidence of AKI in ViV‐TAVR was 6.95% (±6%) and in redo SAVR was 15.2% (±9.6%). For RRT, our data showed that VIV‐TAVR to be 1.48% (±1.46%) and redo SAVR to be 8.54% (±8.06%).

**Conclusion:**

Renoprotective strategies should be put into place to prevent and reduce AKI incidence regardless of the treatment modality. Patients undergoing re‐intervention for the aortic valve constitute a high‐risk and frail population in which ViV‐TAVR demonstrated it might be a feasible option for carefully selected patients. Long‐term follow‐up data and randomized control trials will be needed to evaluate mortality and morbidity outcomes between these 2 treatments.

## 1 INTRODUCTION

Aortic stenosis constitutes the single most common primary valve pathology, regularly requiring either surgical or transcatheter intervention. Over the last decade, a significant drive toward the utilization of bioprosthetic aortic valves has been observed, especially among younger patients prioritizing the avoidance of lifelong anticoagulation and the associated increased quality of life.^1^ Nevertheless, the durability of bioprosthetic valves remains limited, with an important number of patients requiring re‐intervention for the failed aortic valve. Currently, redo surgical aortic valve replacement (SAVR) remains the gold standard treatment for the failed bioprosthetic aortic valve. However, redo SAVR brings potentially major difficulties associated both with the technical complexity of the operation and with the advancing comorbidities and age of the patients. Transcatheter valve‐in‐valve aortic valve replacement (ViV‐TAVR) represents a relatively recent therapeutic option used for the treatment of high or prohibitive surgical risk patients. ViV‐TAVR offers a less invasive alternative avoiding repeat sternotomy in patients and demonstrating acceptable short‐term outcomes.

Postoperative acute kidney injury (AKI) and the requirement for renal replacement therapy (RRT) remain common and significant complications of both treatment modalities.^2^, ^3^ Patients presenting with severe aortic stenosis are often of more advanced age and carry multiple comorbidities including chronic kidney disease (CKD). Nevertheless, the understanding of renal outcomes in the population undergoing either redo SAVR or ViV‐TAVR remains controversial. On the one hand, AKI is a well‐recognized complication in cardiac surgery, being associated with a worse shorter‐ and longer‐term prognosis.^2^ On the other hand, the preoperative and operative stages of TAVR (including the exposure to contrast) could notoriously be associated with AKI in up to 50% of the patients.^4^ Therefore, with the increasing deployment of ViV‐TAVR in the current era, it remains of utmost importance to assess the outcomes related to kidney function between redo SAVR and ViV‐TAVR.

Recently, various meta‐analyses comparing ViV‐TAVR to redo SAVR have been published in the literature. Nevertheless, all of them fell short of reporting renal outcomes, except for Ahmed et al.^5^ which only included AKI results from seven studies, and did not analyze RRT. Furthermore, no studies discussed the implications and role of renal outcomes among the two treatments. The largest and most recent original study to date, by Majmundar et al.^6^ was not included in any of the meta‐analyses. Therefore, this systematic review with meta‐analysis aims to evaluate the evidence in the literature on postoperative renal outcomes, short‐term mortality, and morbidity between redo SAVR and ViV‐TAVR.

## 2 METHODS

### 2.1 Literature search strategy

A systematic review and meta‐analysis were conducted in accordance with the Cochrane Collaboration published guidelines and the Preferred Reporting Items for Systematic Reviews and Meta‐Analyses (PRISMA) statement. A literature search was conducted of EMBASE, MEDLINE, Cochrane, PubMed, and Google Scholar from inception to September 2021 (Supporting Information: Figure 7). The search terms used were: (“transcatheter aortic valve implantation” OR “transcatheter aortic valve replacement” OR “TAVI” OR “TAVR” OR “valve in valve transcatheter replacement” OR “valve in valve transcatheter implantation” OR “ViV‐TAVR” OR “ViV‐TAVI”) AND (“redo surgical aortic valve replacement” OR “Redo SAVR” OR “redo aortic valve surgery”). Further articles were identified through the use of the “related articles” function on MEDLINE and a manual search of the references lists of articles found through the original search. The only limits used were English language and the mentioned time frame. Patient consent and IRB approval were not necessary for this study as no patients were deployed.

### 2.2 Study inclusion and exclusion criteria

All original comparative articles of patients undergoing ViV‐TAVR or redo SAVR for failed aortic valve bioprosthesis. Studies were excluded from the review if: (1) inconsistencies in the data precluded valid extraction; (2) the study was performed in an animal model; (3) studies did not have a comparison group; (4) the size of the study population was small (<10 patients). Case reports, reviews, abstracts from meetings, and preclinical studies were excluded. By using the following criteria two reviewers (A. A. R. and R. V.) independently selected articles for further assessment after the title and abstract review. Disagreements between the two reviewers were resolved by a third independent reviewer (V. N.). Potentially eligible studies were then retrieved for full‐text assessment. Studies presenting overlapping data from the same databases were also excluded (only Hirji et al.^7^).

### 2.3 Data extraction and critical appraisal

All full texts of retrieved articles were read and reviewed by two authors (A. A. R. and R. V.) and inclusion or exclusion of studies were decided unanimously. When there was disagreement, a third reviewer (V. N.) made the final decision. Using a pre‐established protocol, the following data were extracted: first author, study type and characteristics, number of patients, population demographics, AKI rate, renal replacement rate, operative mortality, 30‐day mortality, stroke, major bleeding, and pacemaker implantation rate. For this review, a data extraction sheet was developed, and pilot‐tested on three randomly selected included studies, whereupon the sheet was refined accordingly. Data extraction was performed by two review authors (A. A. R. and R. V.). A third author (V. N.) validated the correctness of the tabulated data. Potential inter‐reviewer disagreements were resolved by consensus. The primary outcomes were AKI and RRT. Secondary outcomes were hospital stroke, pacemaker implantation rate, major bleeding, operative mortality, and in‐hospital mortality.

### 2.4 Data analysis

Odds ratios (OR) with 95% confidence interval (CI) and *p*‐values for 30‐day mortality, operative mortality, AKI, postoperative dialysis, stroke, major bleeding, and pacemaker implantation rates were calculated. Forest plots were created to represent the clinical outcomes. *χ* ^2^ test and *I* ^2^ test were executed for the assessment of statistical heterogeneity. By using a Mantel‐Haenszel random‐effects model, the OR were combined across the studies. Funnel plots were constructed to assess publication bias. All analyses were completed through “metafor” package of R Statistical Software (version 4.0.2; Foundation for Statistical Computing). A two‐tailed *p*‐value <.05 was considered statistically significant. Meta‐regression analyses were performed to investigate the effects of covariates (age, sex, Euroscore, Diabetes, peripheral artery disease, and preoperative chronic kidney disease) on the occurrence of AKI, dialysis, major bleeding, stroke, and operative mortality. Statistical analyses were conducted using the Stata 13.0 software (Stata Corp.).

### 2.5 Sensitivity analysis

The influence of a single study on the overall effect of VIV‐TAVR versus redo SAVR on the main outcome was assessed by sequentially removing one study (the “leave‐one‐out” method). This sensitivity analysis was carried out to test the consistency of these results to investigate if individual studies had an excessive impact on the results.

## 3 RESULTS

### 3.1 Description of studies

The literature search identified 5435 articles. Of these, 115 relevant articles were read in full and assessed according to our inclusion and exclusion criteria. Following the critical appraisal, a total of 18 studies^6^, ^8^, ^9^, ^10^, ^11^, ^12^, ^13^, ^14^, ^15^, ^16^, ^17^, ^18^, ^19^, ^20^, ^21^, ^22^, ^23^, ^24^ incorporating a total of 11,198 patients were included. The studies described outcomes of patients undergoing either ViV‐TAVR (5676 patients) or redo SAVR (6322 patients). Supporting Information: Figure 7 illustrates the study selection process. All the studies included were retrospective nonrandomized studies, with eight of them being multicentre (Supporting Information: Table 1).

### 3.2 Baseline characteristics

Baseline characteristics of the patients included in the studies are summarized in Table 1. The mean age of the patients in the ViV‐TAVR and redo SAVR groups were 76.1 ± 3.3 and 69.7 ± 6.4 years respectively. 62.3% (±10.9) of the patients in the ViV‐TAVR group and 62.6% (±11.8) of the patients in the redo SAVR group were male. Diabetes was present in 27% (±13.7) of patients in the redo SAVR group and 32.9% (±16.4) of the patients in the ViV‐TAVR group. Hypertension and peripheral artery disease (PAD) stood at 77.9% (±7.8) and 16.3% (±12.9) in the redo SAVR group, and at 84.6% (±12.9) and 26.8% (±17.3) in the ViV‐TAVR group, respectively. Pre‐existing acute or chronic renal disease existed in 17.2% (±19.3) of the patients in the redo SAVR group and in 29.4% (±26.4) of those in the ViV‐TAVR group. Five studies reported preoperative creatinine level values with a mean value of 1.11 mg/dl (±0.42) in the redo SAVR group and 1.44 mg/dl (±0.92) in the VIV‐TAVR group.

**Table 1:** Baseline characteristics of patients undergoing ViV‐TAVR versus redo SAVR

Author	Year	Diabetes (%)		Hypertension (%)		PAD (%)		AKI/CKD (%)		Creatinine (mg/dl)		Risk score	
		Redo SAVR	ViV‐TAVR	Redo SAVR	ViV‐TAVR	Redo SAVR	ViV‐TAVR	Redo SAVR	ViV‐TAVR	Redo SAVR	ViV‐TAVR	Redo SAVR	ViV‐TAVR
Erlebach	2015	10	20	73	82	6	10	0	2	1.1 ± 0.3	1.5 ± 1.5	ES 14.4 ± 10	ES 27.4 ± 18.7
Ejiofor	2016	24.6	45.5	81.2	95.5	13	27.3	8.7	27.3	1.10 ± 0.4	1.24 ± 0.5	STS 4.36 ± 3.1	STS 7.54 ± 3.0
Silaschi	2016	10.2	11.3	NA	NA	14	53	NA	NA	1.2 ± 0.4	1.5 ± 1.3	ES 25.1 ± 18.9	ES 16.8 ± 9.3
Grubitzsch	2017	NA	NA	NA	NA	NA	NA	16	59	NA	NA	ES 8.9 ± 6.5	ES 13 ± 10.4
Spaziano	2017	12	20	64	72	10	12	NA	NA	NA	NA	ES 4.4 ± 4.4	ES 7.4 ± 4.9
Sartarpino	2018	62.5	83.3	NA	NA	NA	NA	37.5	33.3	NA	NA	ES 36.4 ± 24.1	ES 33.8 ± 13.8
Seedek	2019	22	28	73	88	14	53	3	1	NA	NA	STS 3.0 (2.1–5.3)	STS 7.5 (4.9–10.7)
Deharo	2020	27.6	28.3	71.7	80.7	32.8	40.2	10.2	21.7	NA	NA	ES2 4.4 ± 1.1	ES2 4.9 ± 1.0
Malik	2020	28.3	32	73	88	28.4	26	17.1	33.1	NA	NA	NA	NA
Stankowski	2020	45	33.8	90	92.5	5	16.1	70	94.1	NA	NA	ES 7.8 ± 4.3	ES 10.9 ± 6.2
Woitek	2020	16.2	36.1	86.5	98	5.4	17.7	7.2	25.2	NA	NA	STS 2.76 ± 2.09	STS 2.76 ± 2.09
Patel	2021	34.9	39	83.7	93.6	NA	NA	3.5	5.9	NA	NA	STS 5.5 ± 4.6	STS 8.4 ± 7.6
Steenbergen	2021	17.6	20.6	NA	NA	NA	NA	NA	NA	NA	NA	ES 15.5 (9.3–22.5)	ES 17.3 (112–23.1
Tam	2021	38.1	43.5	82.6	94.4	4.9	10.2	14.5	12.1	NA	NA	NA	NA
Vukadinovikj	2021	NA	NA	NA	NA	NA	NA	NA	NA	0.9 ± 0.1	1.4 ± 0.1	ES 17.7 ± 4.6	ES 26.8 ± 3.1
Dokollari	2021	28.1	22.6	82.5	77.4	38.6	51.6	NA	NA	1.27 ± 0.92	1.58 ± 1.21	ES2 11.02 ± 9.33	ES2 9.46 ± 7.3
Choi	2021	27	26	68	50	4	5.48	NA	NA	NA	NA	ES 4.32 ± 2.98	ES 7.51 ± 8.24
Majmudar	2021	29.7	36.3	83	88.3	36.3	25.8	18.9	38	NA	NA	NA	NA

Abbreviations: AKI, acute kidney injury; CKD, chronic kidney disease; PAD, peripheral artery disease; SAVR, surgical aortic valve replacement; ViV‐TAVR, valve‐in‐valve aortic valve replacement.

### 3.3 Primary outcomes

#### 3.3.1 AKI

**Figure 1:**
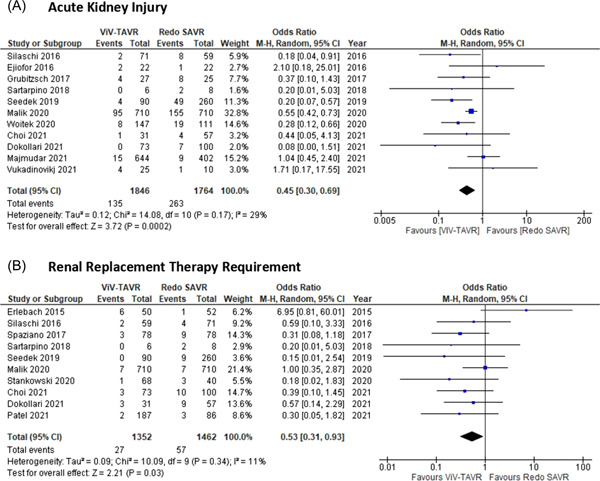
Forrest plots. Pooled odds ratio and conclusions plot for (A) acute kidney injury and (B) renal replacement therapy requirements.

ViV‐TAVR was compared to redo SAVR with 11 studies^6^, ^10^, ^14^, ^15^, ^16^, ^17^, ^18^, ^19^, ^21^, ^22^, ^24^, ^25^ reporting on AKI outcomes postoperatively (Figure 1A). The overall OR for AKI showed a statistically significant difference in favor of ViV‐TAVR versus redo SAVR (random‐effects model: OR: 0.45; 95% CI: 0.30–0.69; *p* < .001). There was evidence of low heterogeneity among studies reporting on AKI.

#### 3.3.2 RRT requirement

ViV‐TAVR was compared to redo SAVR with 10 studies^8^, ^9^, ^11^, ^15^, ^16^, ^18^, ^20^, ^21^, ^22^, ^24^ reporting on patients requiring postoperative RRT (Figure 1B). The overall OR for RRT showed a statistically significant difference favoring ViV‐TAVR over redo SAVR (random‐effects model: OR: 0.53; 95% CI: 0.31–0.93; *p* = .03). There was evidence of low heterogeneity among studies reporting on postoperative RRT.

### 3.4 Secondary outcomes

#### 3.4.1 Major bleeding

**Figure 2:**
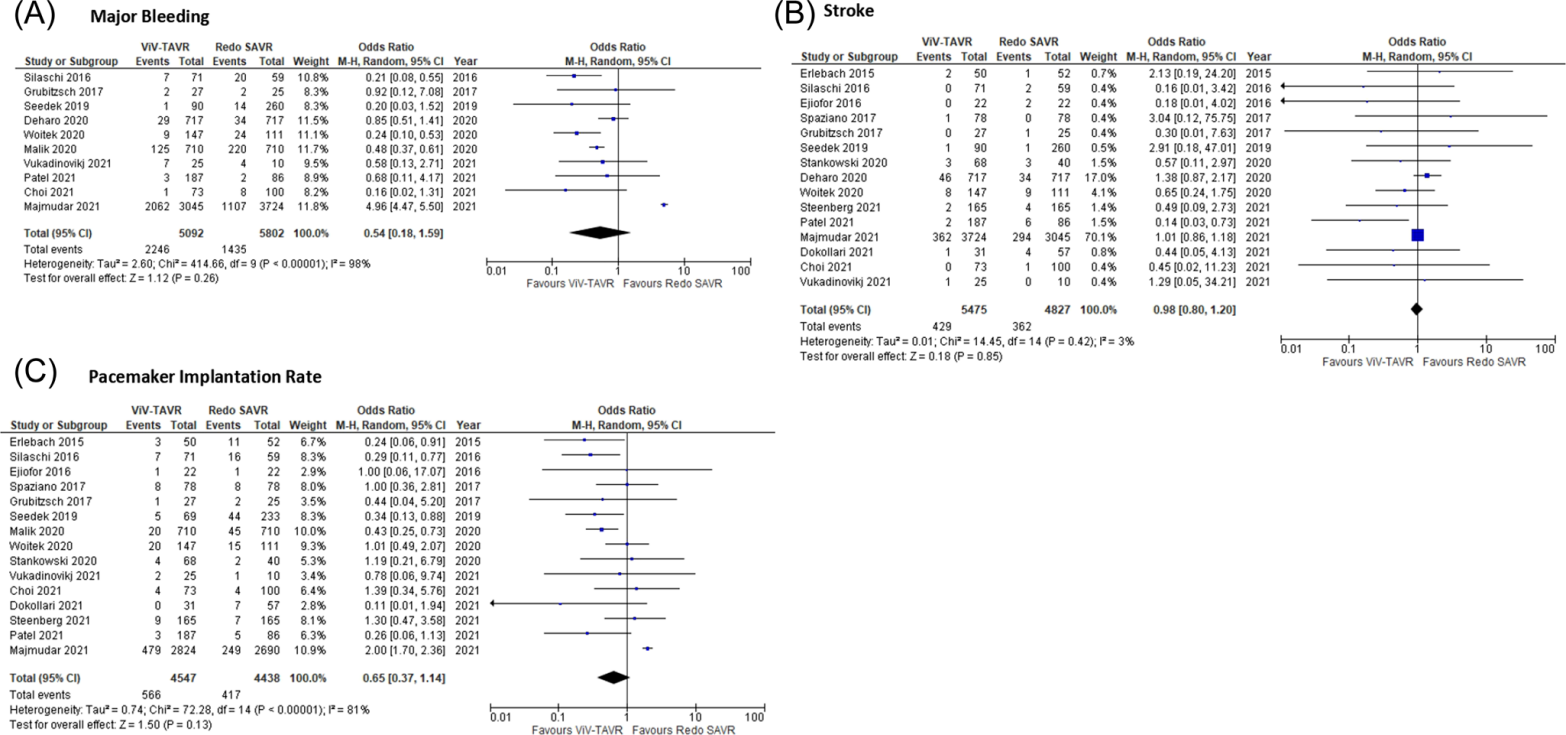
Forrest plots. Pooled odds ratio and conclusions plot for (A) major bleeding, (B) stroke, and (C) pacemaker implantation.

ViV‐TAVR was compared to redo SAVR in 10 studies^6^, ^10^, ^11^, ^14^, ^16^, ^18^, ^19^, ^22^, ^23^, ^24^ reporting on major bleeding outcomes postoperatively (Figure 2A). The overall OR for major bleeding showed no statistically significant difference between ViV‐TAVR and redo SAVR (random‐effects model: OR: 0.54; 95% CI: 0.18–1.59; *p* = .26). There was evidence of high heterogeneity among studies reporting on major bleeding.

#### 3.4.2 Stroke

ViV‐TAVR was compared to redo SAVR in 15 studies^6^, ^8^, ^9^, ^10^, ^11^, ^12^, ^14^, ^15^, ^16^, ^17^, ^18^, ^19^, ^20^, ^22^, ^23^ reporting on stroke outcomes postoperatively (Figure 2B). The overall OR for stroke showed no statistically significant difference between ViV‐TAVR and redo SAVR (random‐effects model: OR: 0.98; 95% CI: 0.80–1.20; *p* = .85). There was evidence of low heterogeneity among studies reporting on stroke.

#### 3.4.3 Pacemaker implantation rate

The overall OR for pacemaker implantation rate (Figure 2C) demonstrated no statistically significant difference between ViV‐TAVR and redo SAVR (random‐effects model: OR: 0.65; 95% CI: 0.37–1.14; *p* = .13) among 15 studies.^6^, ^8^, ^9^, ^10^, ^11^, ^12^, ^14^, ^15^, ^16^, ^17^, ^18^, ^19^, ^20^, ^22^, ^23^ There was evidence of high heterogeneity among studies reporting on pacemaker implantation rate.

#### 3.4.4 Operative mortality and 30‐day mortality

The overall OR for operative mortality (Supporting Information: Figure 8A) demonstrated a statistically significant difference favoring ViV‐TAVR over redo SAVR (random‐effects model: OR: 0.37; 95% CI: 0.27–0.49; *p* < .001).^6^, ^9^, ^12^, ^14^, ^15^, ^17^, ^18^, ^19^, ^22^, ^24^ There was no evidence of heterogeneity among studies reporting on operative mortality. The overall OR for 30‐day mortality (Supporting Information: Figure 8B) showed no statistically significant difference between ViV‐TAVR and redo SAVR (random‐effects model: OR: 0.92; 95% CI: 0.53–1.60; *p* = .78).^6^, ^8^, ^9^, ^10^, ^11^, ^12^, ^14^, ^16^, ^18^, ^19^, ^20^, ^21^, ^23^ There was evidence of moderate heterogeneity among studies reporting on 30‐day mortality.

### 3.5 Risk for bias across studies

The funnel plot analysis (Supporting Information: Figures 1–6) disclosed an asymmetry around the axis for the operative mortality outcome. No asymmetry around the axis in any of the other outcomes was illustrated through the funnel plots, thus making publication bias related to all outcomes but operative mortality unlikely.

### 3.6 Meta‐regression analysis: Influence of covariates on outcomes

Meta‐regression did not identify a statistically significant influence of covariates on the primary outcomes, namely AKI and postoperative dialysis (Table 2). Similarly, the rate of stroke and operative mortality was also found not to be significantly influenced by any of the analyzed covariates. The rate of major bleeding was found to be significantly influenced by preoperative diabetes (coefficient: −0.08; 95% CI: −0.09 to −0.03; *p* = .022), PAD (coefficient: −0.06; 95% CI: −0.1 to −0.02; *p* = .017) and preoperative CKD (coefficient: −0.11; 95% CI: −0.18 to −0.03; *p* = .013) (Table 2) (Supporting Information: Figures 9–11).

**Table 2:** Meta‐regression analysis: Influence of covariates on outcomes

	Coefficient	Standard error	95% CI	*p* Value
Post‐op AKI				
Age (*n* = 11)	0.12309	0.0654351	−0.0278037 to 0.2739837	.097
Male (*n* = 11)	−0.0129836	0.024954	−0.0705276 to 0.0445605	.617
PAD (*n* = 8)	0.0115632	0.0176955	−0.0339246 to 0.057051	.542
Pre‐op CKD (*n* = 7)	−0.0147088	0.0139468	−0.0534312 to 0.0240136	.351
EuroScore (*n* = 6)	0.023552	0.032	−0.0992199 to 0.0521159	.486
Dialysis				
Age (*n* = 10)	0.0893851	0.0454775	−0.0218944 to 0.2006646	.097
Male (*n* = 10)	−0.0219098	0.0189968	−0.0683932 to 0.0245736	.293
Diabetes (*n* = 10)	0.0139794	0.0151708	−0.0231421 to 0.051101	.392
PAD (*n* = 8)	−0.0216124	0.0100641	−0.0495547 to 0.00633	.098
EuroScore (*n* = 6)	0.0341676	0.0213629	−0.0207475 to 0.0890826	.171
Pre‐op CKD (*n* = 6)	0.0137657	0.0132601	−0.0230502 to 0.0505816	.358
Major bleeding				
Age (*n* = 10)	−0.0571362	0.0490706	−0.1702932 to 0.0560207	.278
Pre‐op CKD (*n* = 7)	−0.1124061	0.0299335	−0.1893527 to −0.0354595	.013
EuroScore (*n* = 5)	0.0242105	0.030477	−0.0503640 to 0.098785	.457
PAD (*n* = 7)	−0.0628425	0.0178437	−0.1087112 to −0.0169737	.017
Diabetes (*n* = 8)	−0.0888205	0.0291428	−0.1601304 to −0.0175106	.023
Male (*n* = 10)	0.0387711	0.0428943	−0.0601432 to 0.1376855	.392
Stroke				
Age (*n* = 15)	−0.0348521	0.0313426	−0.1025637 to 0.0328594	.286
Male (*n* = 15)	0.0303552	0.0365061	−0.0485114 to 0.1092218	.421
Diabetes (*n* = 13)	0.012526	0.027529	−0.0480648 to 0.0731169	.658
PAD (*n* = 11)	−0.0126911	0.0128772	−0.0418212 to 0.0164391	.350
EuroScore (*n* = 10)	0.0373028	0.0508473	−0.0734839 to 0.1480895	.477
Pre‐op CKD (*n* = 9)	0.0035692	0.0116496	−0.0239777 to 0.0311162	.768
Operative mortality				
Age (*n* = 10)	−0.1012102	0.070228	−0.2576879 to 0.0552675	.180
Male (*n* = 10)	−0.0342991	0.0431513	−0.1304462 to 0.0618479	.445
Diabetes (*n* = 9)	0.012526	0.027529	−0.0480648 to 0.0731169	.658
PAD (*n* = 7)	−0.0145955	0.0248926	−0.0755055 to 0.0463144	.579
EuroScore (*n* = 7)	−0.0338821	0.0311498	−0.1043478 to 0.0365836	.305
Pre‐op (*n* = 7)	0.0024354	0.0184232	−0.0449229 to 0.0497937	.900

Abbreviations: AKI, acute kidney injury; CKD, chronic kidney disease; n, number of studies included in the analysis; PAD, peripheral artery disease.

### 3.7 Sensitivity analysis

The sensitivity analysis performed on all outcomes by removing each individual study from the meta‐analysis demonstrated that no single study significantly impacted the OR for AKI, operative mortality, and stroke rate.

#### 3.7.1 RRT

**Figure 3:**
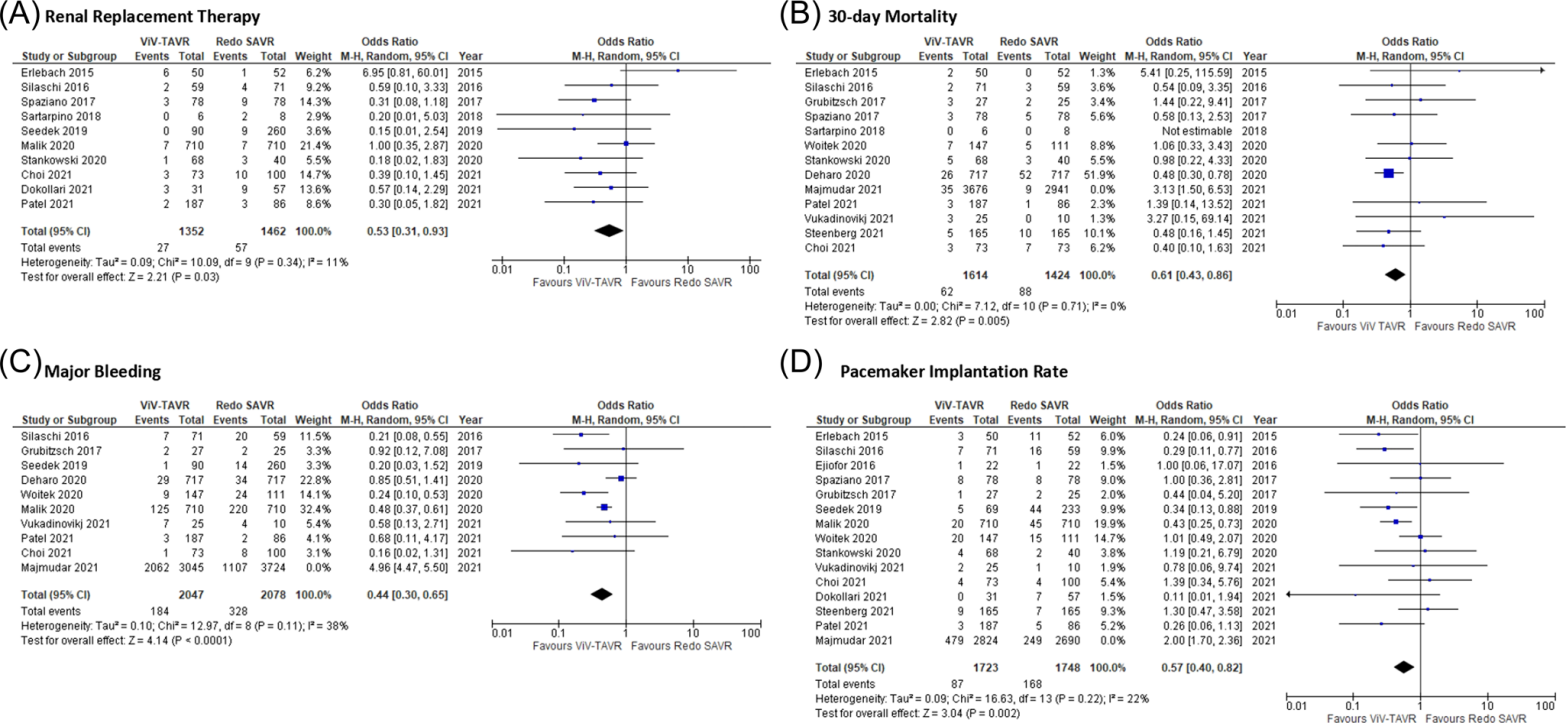
Sensitivity analysis Forrest plots. Pooled odds ratio and conclusions plot for (A) renal replacement therapy, (B) 30‐day mortality, (C) major bleeding, and (D) pacemaker implantation.

For postoperative RRT, after the removal of Erlebach et al.,^8^ the overall OR demonstrated a significant difference favoring ViV‐TAVR over redo SAVR (random‐effects model: OR: 0.47; 95% CI: 0.27–0.79; *p* = .005) (Figure 3A), also reporting no evidence of heterogeneity.

#### 3.7.2 30‐day mortality

For 30‐day mortality, after removal of Majmundar et al.,^6^ the overall OR demonstrated a significant difference favoring ViV‐TAVR over redo SAVR (random‐effects model: OR: 0.61; 95% CI: 0.43–0.86; *p* = .005) (Figure 3B), also reporting no evidence of heterogeneity.

#### 3.7.3 Pacemaker implantation

For permanent pacemaker implantation, after the removal of Majmudar et al.,^6^ the overall OR demonstrated a significant difference favoring ViV‐TAVR over redo SAVR (random‐effects model: OR: 0.52; 95% CI: 0.40–0.82; *p* = .002) (Figure 3C), also reporting evidence of low heterogeneity.

#### 3.7.4 Major bleeding

For major bleeding, after the removal of Majmundar et al.,^6^ the overall OR demonstrated a significant difference favoring ViV‐TAVR over redo SAVR (random‐effects model: OR: 0.44; 95% CI: 0.30–0.65; *p* < .001) (Figure 3D), also reporting evidence of low heterogeneity.

## 4 DISCUSSION

### 4.1 Summary of evidence

To our knowledge, this is the largest meta‐analysis of comparative studies performed to date focussing on ViV‐TAVR versus redo SAVR and the first with a particular focus on postoperative renal outcomes.

In the following meta‐analysis of 18 studies (11,198 patients), we found ViV‐TAVR to be associated with lower rates of AKI, postoperative RRT, major bleeding, pacemaker implantation, operative mortality, and 30‐day mortality. No significant difference between ViV‐TAVR and redo SAVR was observed in terms of stroke rate. Metaregression of covariates (age, sex, Euroscore, Diabetes, peripheral artery disease, and preoperative chronic kidney disease) did not identify a statistically significant influence of covariates on AKI and postoperative dialysis (Table 2). It is important to note that due to the lack of data it was not possible to analyze other desirable postoperative renal outcomes such as urine output, creatinine levels, and duration/modality of RRT.

### 4.2 AKI and RRT

AKI represents a well‐recognized complication in both SAVR and TAVR. Indeed, irrespective of the treatment modality chosen AKI has been found to be a strong predictor for both worsened short‐ and long‐term mortality and morbidity.^2^, ^3^, ^26^ Furthermore, the longer length of stay and higher costs have been associated with this serious complication. In landmark randomized clinical trials comparing TAVR to SAVR, the incidence of AKI has been found to range between 1% to 2% for TAVR and 5% for SAVR.^27^, ^28^, ^29^ Nevertheless, the incidence of AKI in real‐world studies has been found to be much higher for both SAVR and TAVR, ranging from around 10% to 20%.^2^, ^30^ Similarly, our results comparing ViV‐TAVR and redo SAVR, have illustrated the mean incidence of AKI to stand at 6.95% (±6%) for ViV‐TAVR and at 15.2% (±9.6%) for redo SAVR. These numbers reflect the increased incidence of both chronic kidney disease and other comorbidities in real‐world patients undergoing these operations as compared to those enrolled in randomized controlled trials. The findings of our analysis demonstrated that ViV‐TAVR was associated with significantly lower rates of AKI when compared to redo SAVR, therefore supporting the results of the previous meta‐analysis and randomized controlled trials comparing simple TAVR to redo SAVR.^31^ The pathophysiology of AKI post aortic valve replacement remains multifactorial and no causative mechanism for this difference between ViV‐TAVR and redo SAVR has yet been found. Nevertheless, numerous possible explanations associated with both technical and patient‐related characteristics could be provided. Indeed, redo SAVR carries a significant surgical trauma to the patient who often has to undergo a redo‐sternotomy. The deployment of cardiopulmonary bypass (CBP) during the operations exposing blood to artificial surfaces has been shown to activate inflammatory cascades which could eventually cause AKI.^2^ Furthermore, CBP could lead to the generation of microemboli which when smaller than 40 μm are not fully filtered, therefore leading to renal capillary damage.^32^ An increase in renal‐reperfusion injury has also been associated with the decrease in renal perfusion pressure and consequent decrease in renal oxygen delivery brought by CBP.^33^

Although ViV‐TAVR has been shown to present with lower AKI rates relative to redo SAVR, it must be noted that the incidence of these complications remains of serious concern in ViV‐TAVR as well. The rates of AKI in TAVR remain comparable with the rate found by our analysis in ViV‐TAVR, with a recent major nationwide study of 1,07,814 TAVR patients showing the AKI incidence to be 10.7%.^34^ The use of contrast agents during TAVR has indeed been found to be a strong predictor for AKI, and especially contrast‐induced nephropathy. Moreover, rapid ventricular pacing leading to hypotension and thromboembolism resulting from catheter movement in a calcified aorta are some of the other ViV‐TAVR‐associated risk factors for AKI development.^33^ The use of a nonfemoral approach during TAVR was also found in multiple studies to be associated with a higher occurrence of AKI.^33^ It is noteworthy that patients currently undergoing ViV‐TAVR tend to be higher risk patients usually deemed unfit for redo SAVR, thus also having increased age and comorbidities when compared to their surgical counterparts. Therefore, it could be argued that as the trend in ViV‐TAVR deployment grows and lower‐risk patients are enrolled, the risks of developing AKI could decrease.

Our analysis also found postoperative RRT requirement to be lower with ViV‐TAVR than with redo SAVR. Data regarding the rates of RRT post‐VIV‐TAVR or redo SAVR remain unclear and vary greatly among studies, our data showed RRT with VIV‐TAVR to be 1.48% (±1.46%) and with redo SAVR to be 8.54% (±8.06%). RRT often constitutes the end spectrum of AKI and has also been associated with poorer short‐ and long‐term mortality, morbidity, and quality of life outcomes. However, it is important to note that patients with CKD often form part of the exclusion criteria for studies. Indeed, Hirji et al.^7^ and Majmundar et al.^6^ constituting the two largest comparative studies in the field both lack data regarding dialysis requirements.

### 4.3 The implications of AKI on healthcare systems and costs

The implications of developing AKI following either ViV‐TAVR or redo SAVR comprise not only a serious issue for patients but could also translate into increased costs and burden for healthcare systems. An analysis^35^ of 1,078,036 cardiac surgical operations carried out in the United States, illustrated that the mean total index costs of hospitalization for cardiac surgery patients developing AKI were almost double than those without AKI ($77,178 vs. $38,168). At a nationwide level, the researchers found the hospitalization cost associated with AKI postcardiac surgery to be $1.01 billion,^35^ illustrating how even a 10% decrease in the rate of AKI could result in massive cost savings for hospitals. Similar results were recently reported in an analysis of the National Inpatient Sample (United States) illustrating that patients with AKI undergoing TAVR were subject to both an increase in the cost of hospital stay ($2,58,056 vs. $1,74,673) and length of stay (9 vs. 3 days).^36^ Although, comparative data regarding the additional costs of AKI development post‐ViV‐TAVR and redo SAVR is lacking, knowledge from studies on TAVR and SAVR underline the massive impact of this complication.

### 4.4 Strategies to reduce the incidence of AKI in ViV‐TAVR and redo SAVR

Reducing the incidence of AKI in patients undergoing ViV‐TAVR or redo SAVR remains of utmost importance. The identification of the numerous procedural and patient‐related risk factors will lead to careful procedural planning and patient selection. Indeed, action to address AKI should take place on multiple fronts. Initially, optimizing renal perfusion preoperatively through adequate patient hydration, inotrope usage, and thorough hemodynamic monitoring should take place, especially for patients with CKD. Nephrotoxic medications discontinuation should also be considered following careful discussion with the Heart Team. Usage of diuretics such as Furosemide, to improve urine output could also be considered. For patients undergoing redo SAVR, although outcomes remain scarce and controversial, Fenoldopam Mesylate (a selective agonist of DA‐1 receptors) could be considered as it has been shown to reduce postoperative AKI incidence.^37^ For patients undergoing ViV‐TAVR several options are present which could help improve outcomes, including the increased use of sedation over general anesthesia and the deployment of renoprotective systems such as *Renal Guard System*, ensuring Furosemide induced diuresis matched with intravenous isotonic hydration.^38^

Contrast has been extensively reported to be a known risk factor for AKI, especially in patients with previously diagnosed renal impairment, therefore, the use of noncontract imaging represents another renoprotective avenue. The following is particularly true for patients with an already implanted aortic valve prosthesis, where noncontrast CT scan and angiographic studies could be facilitated by the presence of the prosthesis or the amount of calcium in the aortic annulus.^39^ Minimal contrast TAVR, with the administration of <10 ml of contrast has also been shown in some studies to be a possible feasible and safe alternative.^40^

### 4.5 Complications in ViV‐TAVR versus redo SAVR

When the study by Majmundar et al.^6^ was excluded in the sensitivity analysis, ViV‐TARV was shown to lead to less episodes of postoperative major bleeding events. Similar to the results of our sensitivity analysis, major trials assessing TAVR versus SAVR have demonstrated that surgery was associated with increased episodes of major bleeding when compared to TAVR. Major bleeding, whether in SAVR or TAVR, has been associated with an increased incidence of AKI, mainly explained due to the probable ischemic damage posed to the kidneys. Transcatheter technologies have drastically evolved over the past decade to include a reduced rate of bleeding and vascular complications, mainly attributed to improvements in the valve designs and reduced sheath sizes. Nevertheless, the study by Majmundar et al.^6^ remains the largest multicentre study published to date, with the results indicating redo SAVR to be superior with regard to major bleeding outcomes when compared to ViV‐TAVR.

In the present study, the stroke rate was also analyzed, demonstrating no statistically significant difference between ViV‐TAVR and redo SAVR. Contrarily, ViV‐TAVR was associated with lower pacemaker implantation rates when compared to redo SAVR. Deharo et al.^23^ analyzed the data of 1434 patients from the French National Database and found similar results with regard to stroke outcomes. Likewise, Majmudar et al.^6^ in their analysis of 6769 procedures, demonstrated both stroke and pacemaker implantation rate to be comparable between redo SAVR and ViV‐TAVR. Similar results were also reported by Malik et al.^24^ in the evaluation of 1420 patients.

### 4.6 Mortality in ViV‐TAVR versus redo SAVR

Hereby, we found that ViV‐TAVR was associated with better operative mortality outcomes when compared to redo SAVR. Redo aortic valve surgery is known to be related to an increased risk of complications and mortality mainly due to the high risk and frail patients who undergo the operation, which often entails a redo‐sternotomy. The results of Majmundar et al.^6^ support the ones of our analysis with regard to operative mortality, illustrating the improved outcomes with ViV‐TAVR. Similarly, Malik et al.^24^ report improved outcomes in ViV‐TAVR with respect to operative mortality. In line with the findings of Malik et al.,^24^ and the findings of the previous meta‐analysis published on the subject, we found a significant difference favoring ViV‐TAVR in terms of 30‐day mortality. Our results contrast the ones of Majmundar et al.^6^ who found both mortality at 30 days and at 6 months to be comparable between the two treatment modalities. Similarly, the previous meta‐analysis by Gozdek et al.^41^ found that although there was no statistical difference in procedural mortality, 30‐day and cardiovascular mortality at a mean follow‐up of 18 months, cumulative survival analysis favored surgery. Nevertheless, the latter meta‐analysis only included four studies with a total of 347 patients and was carried out at the early stages of ViV‐TAVI deployment, thus probably reflecting the older age and higher risk of ViV‐TAVR who were mostly “non‐reoperable” using surgical techniques. During the last few years, it has been clear that in light of the improved outcomes, the off‐label uses of ViV‐TAVR as well as the number of procedures carried out have been increasing, thus probably explaining our results. Considering the general higher risk population in terms of age and comorbidities which undergoes ViV‐TAVR, it could be suggested that ViV‐TAVR could be safely performed in carefully selected patients. Nevertheless, it is important to note that long‐term mortality and morbidity data comparing ViV‐TAVR to redo SAVR is currently missing, therefore leaving redo SAVR as the mainstay treatment.

### 4.7 Limitations

Although our study provides considerable evidence with regard to both short‐term clinical outcomes and renal outcomes comparing ViV‐TAVR to redo SAVR, there are several limitations in both our study design and reporting of outcomes which impact the interpretation of our results. First, it was not possible to carry out a cluster analysis taking into consideration the different types of valves deployed in both redo SAVR and ViV‐TAVR. Similarly, due to the lack of individual patient data, a comparison based on the pathology of the aortic valve and the grade of AKI was not possible. Heterogeneity remains a further issue of consideration, as patients assigned to ViV‐TAVR tend to be older and have more comorbidities than their redo SAVR counterpart. Even in larger retrospective multicentre studies, the lack of long‐term follow‐up data limits our ability to understand the long‐term performance of the two treatments. Lastly, due to a lack of patient data, it was not possible whether renoprotective strategies were put into place or not.

## 5 CONCLUSION

AKI and the need for RRT both constitute a serious and prevalent complication in both ViV‐TAVR and redo SAVR. Therefore, renoprotective strategies should be put into place to prevent and reduce AKI incidence regardless of the treatment modality. Patients undergoing re‐intervention for the aortic valve constitute a high‐risk and frail population in which ViV‐TAVR demonstrated a lower incidence of AKI, RRT, major bleeding, pacemaker implantation, operative mortality, and 30‐day mortality. Stroke rates remain unchanged between ViV‐TAVR and redo SAVR, demonstrating that ViV‐TAVR might be a feasible option for carefully selected patients. Long‐term follow‐up data and randomized control trials will be needed to evaluate mortality and morbidity outcomes between these two treatments.

## CONFLICT OF INTEREST

The authors declare no conflict of interest.

## Supporting information

Supporting information.
