# Supplementary material for: Renal outcomes in valve‐in‐valve transcatheter versus redo surgical aortic valve replacement: A systematic review and meta‐analysis
Source: J Card Surg. 2022 Aug 30;37(11):3743–53. doi: 10.1111/jocs.16890 (PMC9804591; doi:10.1111/jocs.16890)
Supplement: Supplementary file 1 — Supporting information. [file JOCS-37-3743-s001.docx]

|  | Year | Study characteristics | Number of patients | | Male (%) | | Age (years) | |
| --- | --- | --- | --- | --- | --- | --- | --- | --- |
|  |  |  | **Redo SAVR** | **ViiV-TAVR** | **Redo SAVR** | **ViV-TAVR** | **Redo SAVR** | **ViV-TAVR** |
| **Erlebach** (8) | **2015** | **R, NM, NR** | **52** | **50** | **73** | **54** | **66.2** | **78.1** |
| **Ejiofor** (9) | **2016** | **R, NM, NR** | **22** | **22** | **59.1** | **63.6** | **74.5** | **75** |
| **Silaschi** (10) | **2016** | **R, M, NR** | **59** | **71** | **61** | **57.7** | **72.9** | **78.6** |
| **Grubitzsch** (11) | **2017** | **R, NM, NR** | **25** | **27** | **77** | **77** | **69** | **75.3** |
| **Spaziano** (12) | **2017** | **R, M, NR** | **78** | **78** | **66** | **50** | **77.4** | **78** |
| **Sartarpino** (13) | **2018** | **R, NM, NR** | **8** | **6** | **25** | **66.7** | **78.8** | **80.2** |
| **Seedek** (14) | **2019** | **R, NM, NR** | **260** | **90** | **68** | **81** | **72** | **79** |
| **Deharo** (15) | **2020** | **R, M, NR** | **717** | **717** | **57.7** | **56.1** | **74.5** | **74.9** |
| **Malik** (16) | **2020** | **R, M, NR** | **710** | **710** | **64.9** | **62.8** | **73.3** | **73.7** |
| **Stankowski** (17) | **2020** | **R, NM, NR** | **40** | **68** | **62.5** | **41.2** | **72.9** | **79.2** |
| **Woitek** (18) | **2020** | **R, NM, NR** | **111** | **147** | **59.9** | **62.9** | **58.5** | **76.2** |
| **Patel** (19) | **2021** | **R, NM, NR** | **86** | **187** | **66.3** | **67.9** | **61.3** | **73** |
| **Steenbergen** (20) | **2021** | **R, M, NR** | **165** | **165** | **60.6** | **58.2** | **74** | **73** |
| **Tam** (21) | **2021** | **R, M, NR** | **131** | **131** | **61.8** | **61.8** | **76.1** | **75.9** |
| **Vukadinovikj** (22) | **2021** | **R, NM, NR** | **10** | **25** | **80** | **80** | **62.9** | **75.4** |
| **Dokollari** (23) | **2021** | **R, NM, NR** | **57** | **31** | **50.9** | **54.8** | **67.19** | **79.1** |
| **Choi** (24) | **2021** | **R, NM, NR** | **100** | **73** | **72** | **75.3** | **58.3** | **66.6** |
| **Majmudar**(25) | **2021** | **R, M, NR** | **3045** | **3724** | **61.1** | **51.1** | **65** | **79** |

**Supplementary File**

**Suppl.Table 1: characteristics studies comparing ViV-TAVR to Redo SAVR**


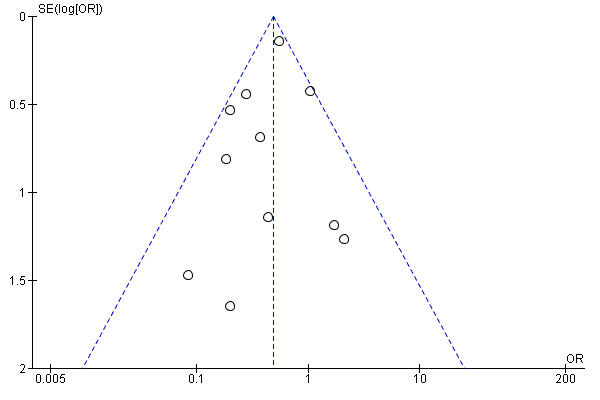


**Suppl.Fig.1 Publication Bias Analysis of Clinical Outcomes by Funnel Plot Graphic for Surgery versus no-surgery groups: AKI**


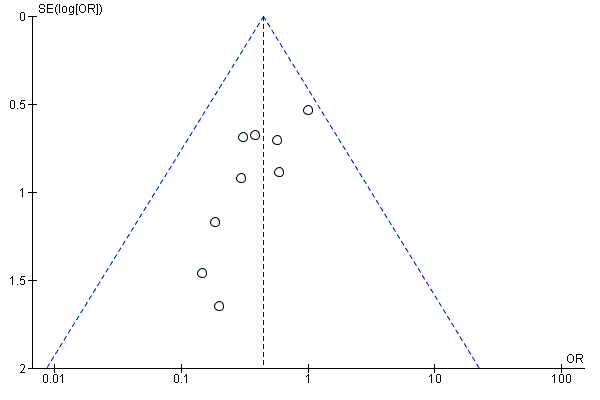


**Suppl.Fig.2 Publication Bias Analysis of Clinical Outcomes by Funnel Plot Graphic for Surgery versus no-surgery groups : Renal Replacement Therapy**


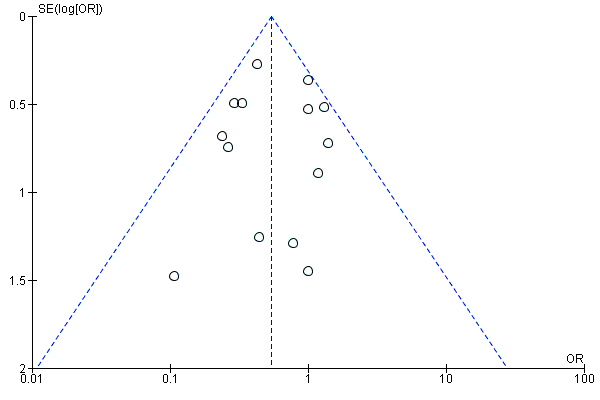


**Suppl.Fig.3 Publication Bias Analysis of Clinical Outcomes by Funnel Plot Graphic for Surgery versus no-surgery groups : Pacemaker implantation rate**


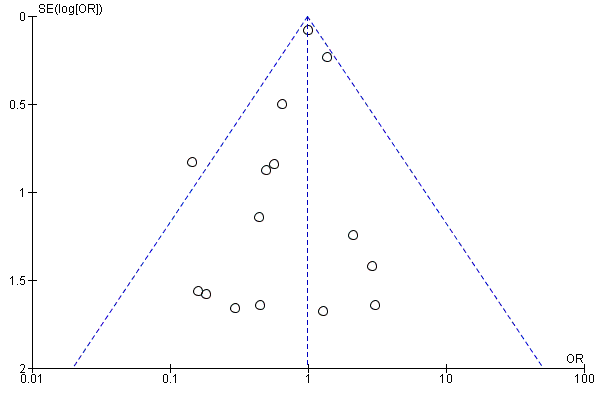


**Suppl.Fig.4 Publication Bias Analysis of Clinical Outcomes by Funnel Plot Graphic for Surgery versus no-surgery groups : Stroke**


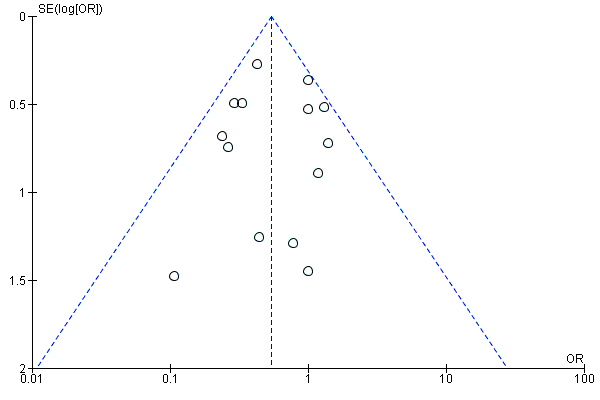


**Suppl.Fig.5 Publication Bias Analysis of Clinical Outcomes by Funnel Plot Graphic for Surgery versus no-surgery groups : Operative Mortality**


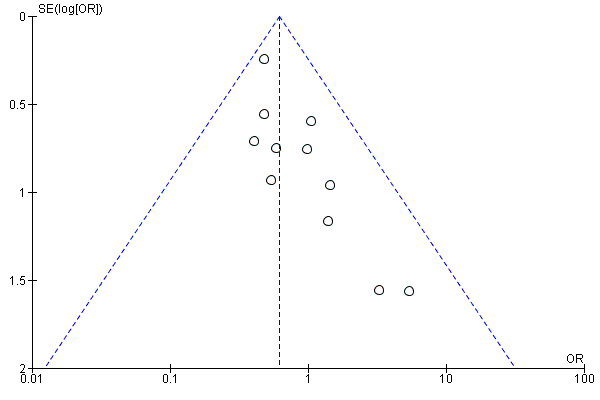


**Suppl.Fig.6 : Publication Bias Analysis of Clinical Outcomes by Funnel Plot Graphic for Surgery versus no-surgery groups: 30-day mortality**


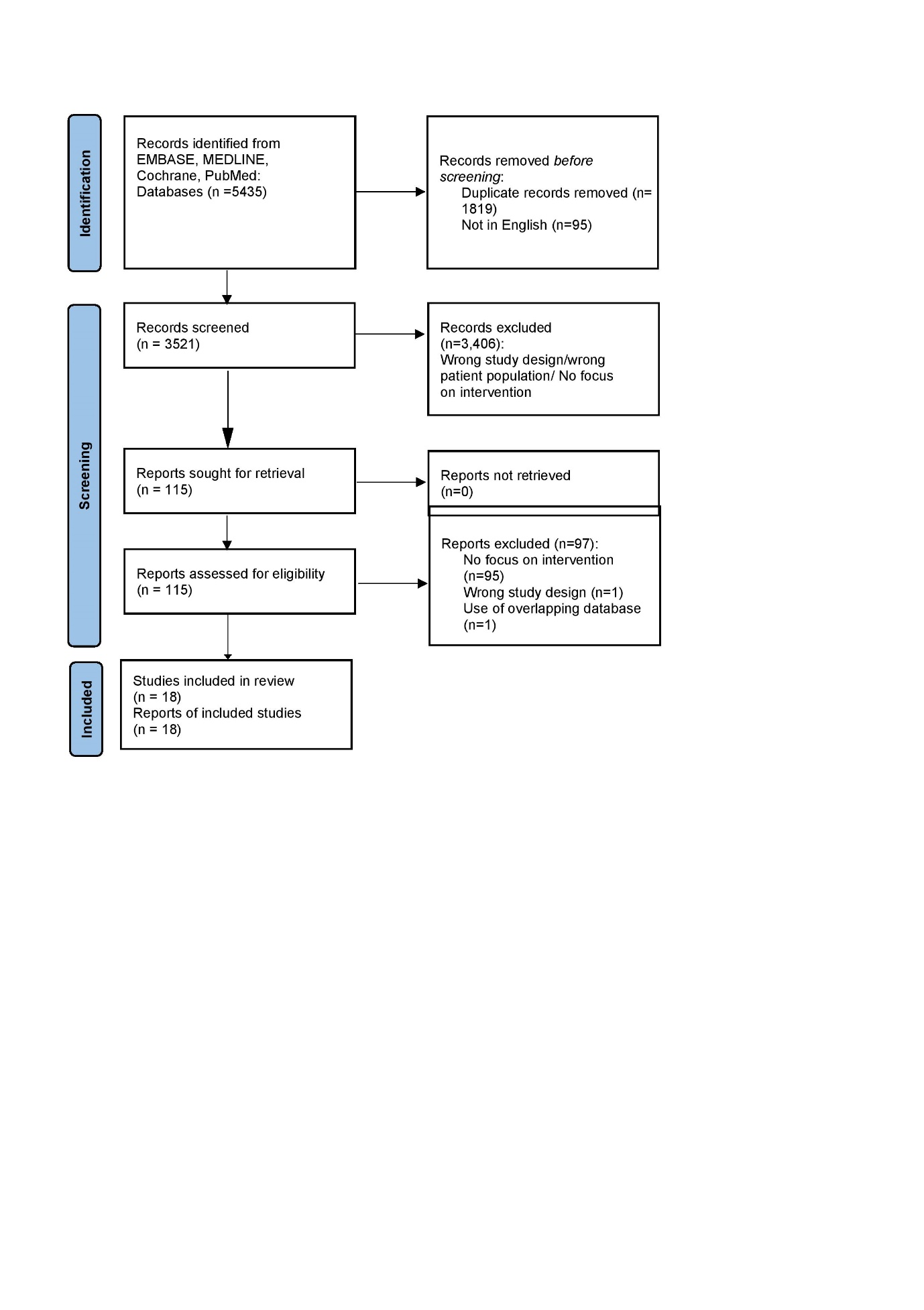


**Suppl.Fig. 7: PRISMA flow diagram**


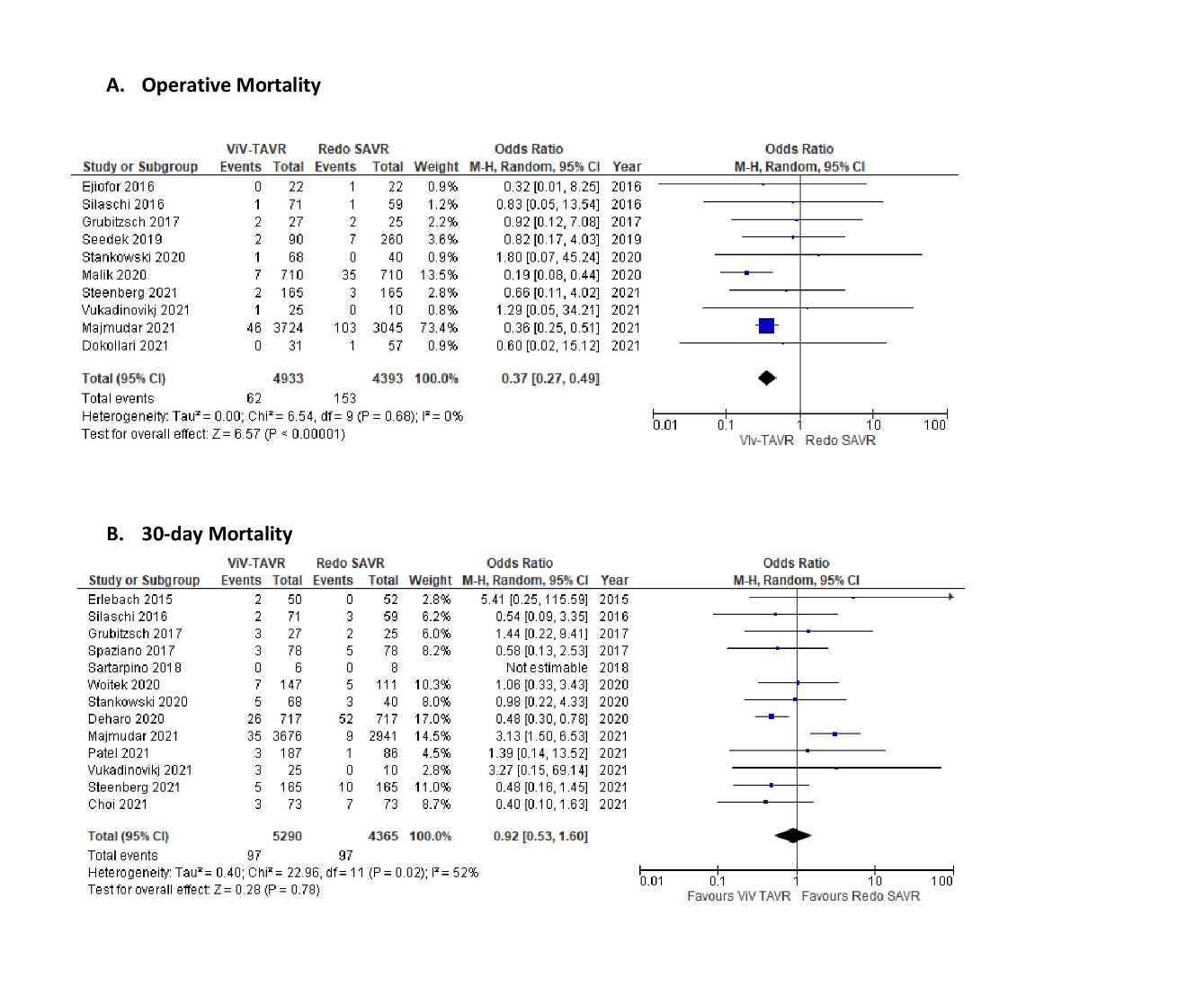


**Suppl.Fig. 8:** Forrest plots. Pooled odds ratio and conclusions plot for (A) Operative Mortality, (B) 30-day mortality.

**Suppl fig. 9: Bubble plot with respect to chronic kidney disease. y-axis represents the log(OR) for re-SAVR vs ViV-TAVI with respect to major bleeding**

**
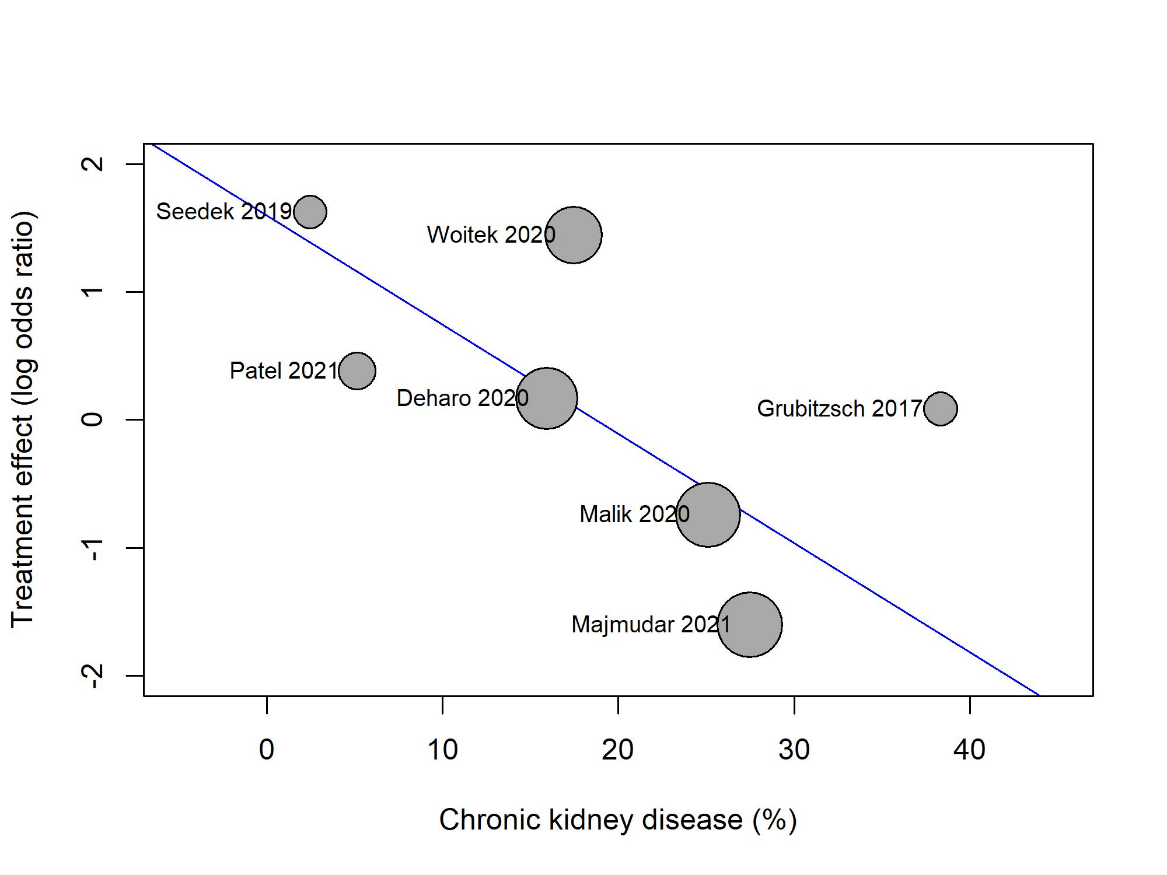
**

**Suppl fig. 10: Bubble plot with respect to diabetes mellitus. y-axis represents the log(OR) for re-SAVR vs ViV-TAVI with respect to major bleeding**

**
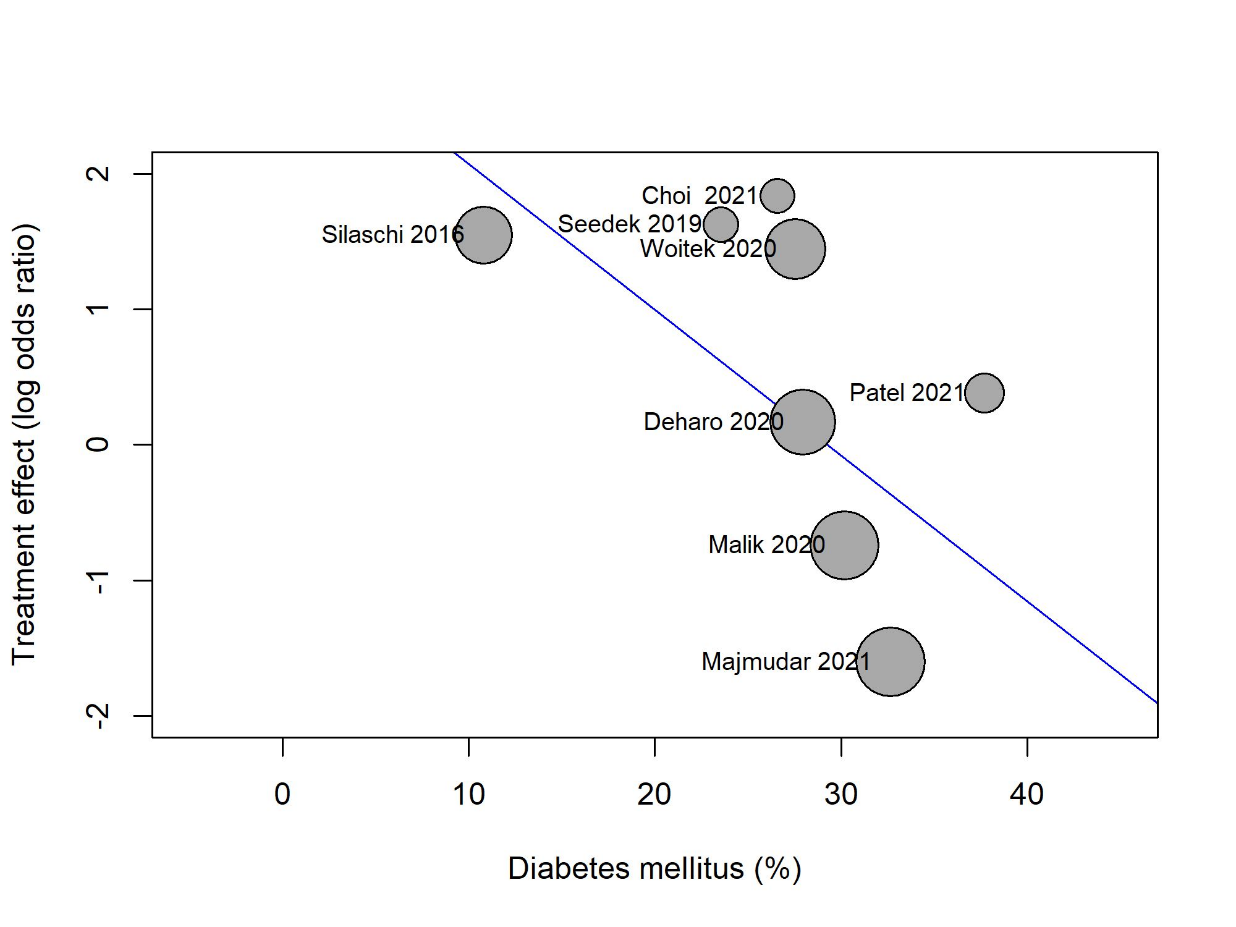
**

**Suppl fig. 11: Bubble plot with respect to peripheral artery disease. y-axis represents the log(OR) for re-SAVR vs ViV-TAVI with respect to major bleeding**

**
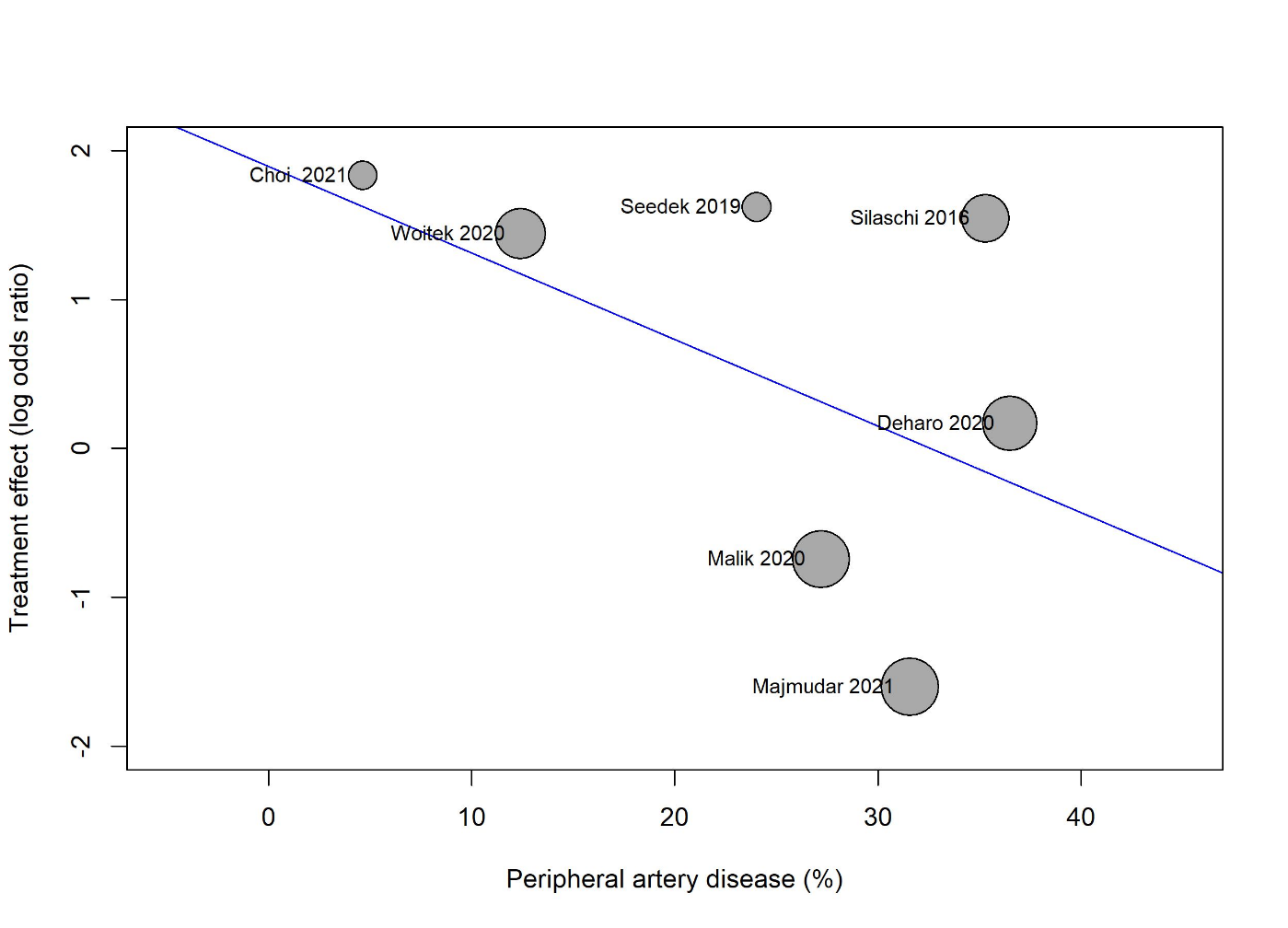
**
